# Crop epigenetics and the molecular hardware of genotype × environment interactions

**DOI:** 10.3389/fpls.2015.00968

**Published:** 2015-11-06

**Authors:** Graham J. King

**Affiliations:** ^1^Southern Cross Plant Science, Southern Cross University, Lismore, NSW, Australia; ^2^National Key Laboratory for Crop Genetic Improvement, Huazhong Agricultural University, Wuhan, China; ^3^Crops for the Future, Biotechnology and Breeding Systems, Semenyih, Malaysia

**Keywords:** crop epigenetics, chromatin dynamics, thermal homeostasis, ionic homeostasis, DNA methylation, phenotypic plasticity, G × E interactions

## Abstract

Crop plants encounter thermal environments which fluctuate on a diurnal and seasonal basis. Future climate resilient cultivars will need to respond to thermal profiles reflecting more variable conditions, and harness plasticity that involves regulation of epigenetic processes and complex genomic regulatory networks. Compartmentalization within plant cells insulates the genomic central processing unit within the interphase nucleus. This review addresses the properties of the chromatin hardware in which the genome is embedded, focusing on the biophysical and thermodynamic properties of DNA, histones and nucleosomes. It explores the consequences of thermal and ionic variation on the biophysical behavior of epigenetic marks such as DNA cytosine methylation (5mC), and histone variants such as H2A.Z, and how these contribute to maintenance of chromatin integrity in the nucleus, while enabling specific subsets of genes to be regulated. Information is drawn from theoretical molecular *in vitro* studies as well as model and crop plants and incorporates recent insights into the role epigenetic processes play in mediating between environmental signals and genomic regulation. A preliminary speculative framework is outlined, based on the evidence of what appears to be a cohesive set of interactions at molecular, biophysical and electrostatic level between the various components contributing to chromatin conformation and dynamics. It proposes that within plant nuclei, general and localized ionic homeostasis plays an important role in maintaining chromatin conformation, whilst maintaining complex genomic regulation that involves specific patterns of epigenetic marks. More generally, reversible changes in DNA methylation appear to be consistent with the ability of nuclear chromatin to manage variation in external ionic and temperature environment. Whilst tentative, this framework provides scope to develop experimental approaches to understand in greater detail the internal environment of plant nuclei. It is hoped that this will generate a deeper understanding of the molecular mechanisms underlying genotype × environment interactions that may be beneficial for long-term improvement of crop performance in less predictable climates.

## Introduction

Crop plants are sessile autotrophs, represented by relatively few monocotyledon and dicotyledon angiosperm species which lack the internal thermoregulation of hot blooded animals. Modern breeding programs have contributed to increases in yield, with major advances made during a period of relative climate stability. However, the planet has entered a period of climate variability, in which higher global temperatures also increase amplitude and temporal variance of climate parameters, and temperature accounts for over 30% of global crop yield variability (Porter and Semenov, 2005; Ray et al., 2015). These effects are compounded by the progressive salinization of many available arable soils (Pimental et al., 2004).

Such issues require a deeper understanding of the molecular mechanisms underlying plant responses to the environment (Baulcombe and Dean, 2014). Crop performance, yield and quality are sensitive to interactions between genotype and environment (GxE), with built-in phenotypic plasticity required for crop cultivars to cope with variable environments (Bloomfield et al., 2014). This is particularly critical where management of the internal thermal and ionic environment affects growth rates and developmental phase transitions. The internal ionic status of a plant is strongly dependent on external nutrient availability, with mineral fertilizers a major cost for crop production (Timilsena et al., 2014). In particular, the major macronutrient potassium plays a key role in metabolic adjustment during plant development, affecting yield and responses to salinity, drought and cold.

The detection of temperature by plants is required for appropriate responses on multiplexed timescales covering periods from seconds to years (Blanchard and Runkle, 2011; Way and Pearcy, 2012), with supply of mineral ions varying on an intermediary timescale (Le Bot et al., 1998). Crop yields are sensitive to the pattern of diurnal variation in air and soil temperature that affects the rate of growth and development (Schlenker and Roberts, 2009; Lobell and Gourdji, 2012; Gourdji et al., 2013). Productivity is dependent both on the ability to perceive minor fluctuations in ambient temperature, as low as ±1°C (Porter and Semenov, 2005; Hüve et al., 2006; Blanchard and Runkle, 2011), and plastic responses that involve keeping tally of accumulated thermal history during specific developmental phases (Muchow et al., 1990; McMaster and Wilhelm, 1997; Tan et al., 2000). Thus fluctuations in thermal environment that perturb the ontogenetic timeline have potential for a significant impact on crop yield and quality (Craufurd and Wheeler, 2009; Bloomfield et al., 2014; Ray et al., 2015).

Although many crop traits and developmental phase changes are dependent upon thermal and ionic signals, conventional genetic models have not provided a complete understanding of the relevant signal transduction pathways and behavior (Hammond et al., 2011; McClung and Davis, 2010). More recently it has become apparent that epigenetic marks play a significant role and are able to provide a mechanistic framework in the context of chromatin dynamics.

Environment is detected in a number of ways, including via effector proteins, small RNAs (Mirouze and Paszkowski, 2011) and directly by chromatin (Kumar and Wigge, 2010). As we will see, sophistication and ruggedness in crop plasticity depends to a great extent upon epigenetic feedback loops that contribute to genomic regulation, with crosstalk between physiological and sub-cellular systems (Bloomfield et al., 2014; Kissoudis et al., 2014; Kulcheski et al., 2015). Whilst many of these mechanisms involve specific gene networks and epigenetic marks, at the molecular level within the plant nucleus the relationship between temperature and electrostatic interactions mediated by ion concentration merits investigation.

Plant growth and development progress within biophysical and thermodynamic constraints imposed by the molecular composition of cells (Lintilhac, 2014; Wolfe, 2015). In eukaryotes, sub-cellular compartmentalization reduces the impact of environment on key sub-systems (Millar et al., 2009), and helps preserve the integrity of enzymes and other informational macromolecules. In the nucleus, the dynamic composition and status of chromatin plays a central role in genomic regulation, and is sensitive to local ionic and thermal environment (Gan and Schlick, 2010; Woodcock and Ghosh, 2010; Allahverdi et al., 2015). Management of temperature and ionic homeostasis represents a major energetic and organizational overhead (Jones and Rotenberg, 2001; Alekseeva et al., 2007; Watling et al., 2008), and involves complex signal transduction systems that are fine-tuned to generate appropriate physiological and developmental responses (Wilson, 2013). These systems include epigenetic processes that provide an environmental memory heritable through mitosis, and in some situations through meiosis (Heard and Martienssen, 2014). Thus it has become clear that RNA-mediated epigenetic mechanisms, along with DNA methylation and histone protein epigenetic marks, significantly extend the adaptive responses of plants (Bräutigam et al., 2013; Pikaard and Scheid, 2014). More complex crop plant genomes, with a high load of repetitive sequences and associated pool of epigenetic marks, may offer greater opportunities for regulation of phenotypic plasticity (Bloomfield et al., 2014).

The performance potential of crop plants relies on maximizing harvest index, the ratio of harvestable to total biomass (Unkovich et al., 2010). This is dependent upon the timely transition between distinct phases of development, where genomic and phenotypic plasticity enables this to be orchestrated in the context of variation in the cultivation environment and crop management practices (Bloomfield et al., 2014). It is recognized that coping with the more extreme environmental fluctuations during a crop cycle is likely to rely on secondary systems (Cramer et al., 2011), as well as gene neo-functionalization, with novel genetic loci conferring distinct regulation or function in order to maintain plant homeostasis (Mickelbart et al., 2015).

This review describes the macromolecular components of the chromatin hardware in which plant genomes are embedded. It explores their biophysical behavior in the context of the ionic and thermal environment of the nucleus, and how this is affected by the local distribution of DNA and histone epigenetic marks. Evidence from *in vitro* and molecular modeling studies is placed where possible in the context of *in vivo* observations for model species and crop plants. The contribution of ions to mediating electrostatic interactions of chromatin and epigenetic marks is placed in the context of ionic variation at whole plant level. Recent advances in understanding how specific epigenetic marks mediate plant thermosensory signaling and other responses to abiotic environment are placed in the context of chromatin dynamics and biophysics.

**FIGURE 1:**
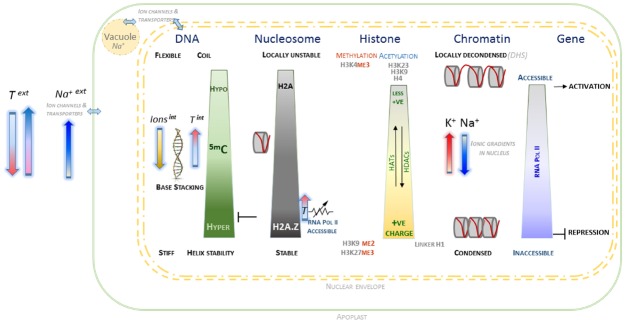
**Schematic overview of interactions associated with chromatin and component macromolecules within the electrostatic environment of the plant nucleus.** The contrasting states of DNA, histones, nucleosomes and chromatin as affected by epigenetic marks and localized nuclear ionic environment are indicated. The arrows indicating external temperature and salt are oriented in relation to their observed effects on DNA methylation and H2A status. Internal cation concentrations contribute to level of chromatin condensation, along with more complex electrostatic interactions, including those involving histone modifications and divalent cations. The contribution of histone acetylases (HAT) and deacetylases (HDAC) to H4 and H3 acetylation are indicated, which along with the interplay with histone methylation is able to provide a complex chromatin code. The nuclear envelope is represented by dashed double yellow lines, with a very simplified representation of interactions between the cytoplasm, vacuole and external apoplast.

A preliminary speculative framework is outlined, based on the evidence of what appears a cohesive set of interactions at molecular, biophysical and electrostatic level between the various components contributing to chromatin conformation and dynamics (Figure 1). It proposes that within plant nuclei, general and localized ionic homeostasis plays an important role in maintaining chromatin conformation, whilst maintaining complex genomic regulation that involve specific patterns of epigenetic marks. More generally, reversible changes in DNA methylation appear to be consistent with the ability of nuclear chromatin to manage variation in external ionic and temperature environment. Whilst tentative, this framework provides scope to develop experimental approaches to understand in greater detail the internal environment of plant nuclei. It is hoped that this will generate a deeper understanding of the molecular mechanisms underlying genotype × environment interactions that may be beneficial for long-term improvement of crop performance in less predictable climates.

## The Genome at Home in the Nucleus

Crop plants have derived from taxa that represent different levels of genome complexity (King, 2002). Some are well adapted to the relatively uniform annual environments of the tropics and subtropics (Gepts, 2008), while others must manage variability in length of temperate seasons and severity of cold winter periods (Rosenzweig and Liverman, 1992; Craufurd and Wheeler, 2009). Compared with the condensed genome of *Arabidopsis* (125 Mbp), crop genome sizes vary over 60-fold, from around 265 Mbp (peach, *Prunus persica*) to the larger cereal genomes (barley, 5.1 Gbp; wheat: 17 Gbp; Michael and Jackson, 2013). In common with all eukaryotes, this variation is directly proportional to nuclear volume (Cavalier-Smith, 1985), which suggests strong selective pressure to maintain an optimal dense nuclear environment, where crowding due to proteins, DNA and RNA leads to a macromolecular concentration calculated at over 100 mg ml^–1^ (Hancock and Hadj-Sahraoui, 2009; Hancock, 2012a), considerably higher than those typically used for many *in vitro* experiments (Hancock, 2012a). This greatly reduced effective solvent volume also means that the equivalent molar concentrations of mono- and divalent ions may be considerably different from those regarded as cytoplasmic or physiological. At present few reliable estimates of nuclear water content exist and, as already noted, it appears that a significant proportion of ions are bound to chromatin and other macromolecules (Garner, 2002; Hancock and Hadj-Sahraoui, 2009).

Compared with the cytoplasm, the rate of molecular mobility, expressed as diffusion constants, is about five times lower in the nucleus, and 10-fold lower in the nucleolus (Bancaud et al., 2009). Despite this, regulatory factors appear still able to locate genomic targets rapidly, due to the presence of electrochemical gradients generated by ion distributions in the remaining space, which have been modeled as channels between chromatin fibers represented by percolation clusters (Wedemeier et al., 2007; Fritsch and Langowski, 2010; Bancaud et al., 2012). Such an environment may also encourage diffusiophoresis (Hancock, 2012a), a process that involves dispersed particles moving spontaneously in a fluid induced by a diffusion or concentration gradient. Indeed, *in vivo* imaging of HeLa nuclei indicates a “mesh spacing” of chromatin fibers on the order of 63 nm, significantly larger than the typical size of diffusing protein complexes (Weidemann et al., 2003). From this and other *in vivo* studies, these authors concluded that all nuclear locations are accessible for diffusing protein complexes (Weidemann et al., 2003). Thus signal transduction via diffusion of regulatory factors to the genome does not appear to be a time-limiting factor for regulatory systems involving rapid transcription, or epigenetic response to external environment. At 7–19 mg ml^–1^, the 30 nM chromatin fiber represents about 10% of the nuclear volume (Strickfaden et al., 2012) and so the time for a non-interacting protein to explore the whole nucleus is of the order of a few seconds, and four times faster than in water (Strickfaden et al., 2012). For example, GFP (27 kDa) may move 12 μM sec^–1^, a similar rate as between the cytoplasm and nucleus (Pack et al., 2006). The extent of this molecular crowding within the nucleus has led to the suggestion that entropic forces, such as those associated with polymer elasticity, may be more significant for chromatin structure and dynamics than some of the electrostatic forces observed under typical *in vitro* conditions (Hancock, 2012b).

## Chromatin Architecture and Dynamics

Eukaryote nuclear chromosomes represent the complex macromolecular structures formed of chromatin, in which genomic DNA is embedded along with highly charged proteins and varying amounts of RNA. Nucleosomes represent the building blocks of chromatin, with ∼147 bp of DNA wrapped around core nucleosome particles, each of which consists of a H3-H3-H4-H4 histone tetramer and two H2A–H2B dimers. Within this octamer, the H2A-H2B dimers occupy the peripheral 2 × ∼30 bp DNA, and the H3–H4 tetramer the inner region, with tails that interact both within and between nucleosomes and contribute to accessibility for transcriptional apparatus (Iwasaki et al., 2013). Post-translational changes to histones, including acetylation and methylation, are able to generate a combinatorial code of epigenetic marks (Lothrop et al., 2013), along with variants such as H2A.Z.

Based on *in vitro* and modeling studies, electrostatic interactions appear to be the dominant factor affecting overall nucleosome stability (Fenley et al., 2010), with higher ionic concentration a major source for destabilization and disruption (Gansen et al., 2009). The wrapping and unwrapping of DNA around the histone core is sensitive to the charge state of the globular core (Fenley et al., 2010), with *in vivo* post-translational acetylation of a single lysine residue in H3 or H4 able to decrease the charge and decrease the strength of binding to DNA. This reduces the tendency of chromatin to fold into highly compact structures, making it more accessible to transcription factors (TFs) and other interacting proteins, as well as having greater sensitivity to DNase I (Garcia-Ramirez et al., 1995; Tse et al., 1998). This epigenetic switch is mediated by histone acetyltransferases (HATs) that neutralize the positive charge on lysine, and histone deacetylases (HDACs) which play an inverse role, by providing a more positive net charge (Eberharter and Becker, 2002).

### Nucleosome Positioning and Epigenetic Marks Define Chromatin State

Nucleosome positioning with respect to genomic sequence is sensitive to many intrinsic and external factors (Tsankov et al., 2011; West et al., 2014), especially in euchromatin, where positioning is dynamic and responsive to cellular identity and internal nuclear environment (Hughes et al., 2012). Gene activation is facilitated by DNA and nucleosome thermodynamics, the nucleosomal surface and chromatin higher order structure (Dechassa and Luger, 2012). Thus initiation and progress of transcription is dependent upon RNA polymerase II (RNA Pol II) gaining access to DNA wrapped around nucleosomes, by harnessing fluctuations that locally unwrap DNA, rather than unwrapping nucleosomes (Mack et al., 2012). Targeted protein and snRNA regulatory factors are able to effect rapid and reversible changes in transcription as a result of this highly dynamic behavior (Ivashuta et al., 2011; Chen and Rajewsky, 2014; Franco-Zorrilla et al., 2014), which is mediated by the epigenetic marks of histone and DNA modifications (Pikaard and Scheid, 2014). The detection of an association between nucleosome phasing, introns, and RNA splicing (Schwartz et al., 2009a; Tilgner et al., 2009; Chodavarapu et al., 2010) also highlights the central role nucleosomes play in managing RNA Pol II transcription in complex eukaryote genomes.

The distribution of epigenetic marks is an important contributor to the organization of nucleosomes in promoter regions, with histone modifications able to mediate very specific access to DNA to enable gene activation (Bannister and Kouzarides, 2011). This can result in distinct and dynamic histone landscapes associated with specific plant processes, such as de-etiolation in *Arabidopsis* (Charron et al., 2009). Experimental data and statistical mechanics thermodynamic predictions both indicate that nucleosomes are able to block the binding of many TFs by competing with common binding sites, as well as contributing to cooperative binding between TFs (Raveh-Sadka et al., 2009). In general, core histones and variant forms tend to be stably bound to DNA on a timescale of hours (Rippe, 2012), whilst the half-life turnover of histone acetylation is on the order of minutes (Nightingale et al., 2007). This confers a combination of high thermodynamic stability whilst being sensitive to factors that allow rapid access to DNA when required.

### Chromatin Accessibility

Decondensed or open chromatin is detectable by the presence of DNAse I hypersensitive sites (DHSs), which provide an accurate experimental indication of where the DNA is exposed and accessible (Wu et al., 2014). For example, in *Arabidopsis* ∼90% of the binding sites of the MADS-box TFs APETALA1 and SEPALLATA3 are covered with DHSs (Zhang et al., 2012), indicating that these form a barrier for nucleosome formation. However, in general only a subset of nucleosomes are reproducibly positioned, with phasing associated with flanking of transcription start sites (TSS) of active genes in *Arabidopsis* (Zhang et al., 2012). In rice, DHSs are associated with regions flanked by strongly phased nucleosome arrays (Wu et al., 2014). This is consistent with a barrier model, where intergenic and other regions in which regulatory proteins may be bound to the genomic DNA to provide a barrier that facilitates phased nucleosome arrays to organize either side (Mobius and Gerland, 2010). Rice DHSs may also span a single phased nucleosome (Wu et al., 2014). For promoters of constitutively transcribed genes a DHS detectable barrier may be permanent (Wu et al., 2014), while for binding sites of TFs associated with tissue-specific, organ-specific or environmentally induced gene expression such barriers may be transient, thus allowing nucleosome rearrangement to facilitate transcription (Zhang et al., 2012).

## Epigenetic Modifications Modify Chromatin Architecture

Although many components of chromatin and epigenetic marks are conserved between plants and animals, it is important to be aware of a number significant plant-specific features. These include 5′-methylcytosine (5mC) occurring in all context in plants compared with solely CG in animals, the presence of hydroxymethylated cytosine (5 hmC) in animals, the distinct plant methyltransferases CMT3, DRM1/2, and MET (Jang et al., 2014) and defined distribution of methylation with respect to *cis*-regulatory and gene body sequences (Cokus et al., 2008). In addition, plants display a characteristic pattern of nucleosome distribution (Chodavarapu et al., 2010), and tighter distribution of intron length (Wu et al., 2013) compared with animals. Plants and animals also appear to have evolved distinct DNA demethylation systems, with the DEMETER (DME) family DNA glycosylases able to remove 5mC efficiently in plants, resulting in DNA demethylation and transcriptional activation of target genes (Jang et al., 2014).

### Management of DNA Methylation

Various experimental approaches have shown that DNA cytosine methylation contributes to regulating higher order chromatin structure in plants (Tariq and Paszkowski, 2004), primarily through interactions with histones, and ultimately affecting nucleosome positioning. There is extensive evidence that gene silencing and repression of active euchromatin is associated with hypermethylation of DNA in plants (Vaillant and Paszkowski, 2007). Although there is less evidence for direct involvement of 5mC in condensation of heterochromatin (Gilbert et al., 2007), at the primary chromatin level DNA methylation has been shown to have a strong interaction with nucleosome formation (Pennings et al., 2005), and particularly in plants (Chodavarapu et al., 2010). These phenomenological observations appear to be consistent with what is known of the underlying properties of the component molecules and their interactions.

5mC has been shown to shift the preferred rotational position of nucleosomes *in vitro* by 3 bp (Buttinelli et al., 1998). In mammals, the stabilizing effect of 5mC on DNA duplexes is able to be reversed by hydroxymethylation to 5 hmC (Rodriguez Lopez et al., 2010; Thalhammer et al., 2011). However, the 5 hmC modification appears absent in plant chromatin (Erdmann et al., 2014), and at present it is unclear whether functional analogs for regulating chromatin dynamics exist.

In plants, the involvement of DNA cytosine methylation (5mC) in regulation of gene expression makes a significant contribution to definition of cellular identity and coordination of ontogeny (Vaillant and Paszkowski, 2007; Heard and Martienssen, 2014). The specific molecular attributes of 5mC compared with unmethylated C, and the dynamic nature of DNA methylation, are critical in providing a “toggle switch” mechanism. Thus 5mC provides a versatile heritable epigenetic mark able to define tissue specific expression patterns, and mediate responses to the environment (Zhang et al., 2011; Bloomfield et al., 2014; Widman et al., 2014). The higher density of 5mCG observed within plant genes compared with promoters (Cokus et al., 2008) appears to have a greater effect on transcription due to inhibition of elongation (Chodavarapu et al., 2010; Gelfman et al., 2013). Within the rice genome, the methylation map indicates single peaks close to start codons (Li et al., 2008). In *Arabidopsis* transcriptional units, 5mC appears to be enriched over the first nucleosome in a transcription unit, with strong periodicity of ∼180 bp in methylation over subsequent nucleosomes (Chodavarapu et al., 2010), and a strong signal associated with positioning in exons.

The processes underlying hypomethylation that are associated with reprogramming, particularly in establishing pluripotency and imprinting effects in plant and animal systems, are now starting to be uncovered (Feng et al., 2010). Dynamic control of DNA methylation involves a cyclic enzyme cascade that consists of cytosine methylation, iterative oxidation of the methyl group by TET (ten eleven translocation) dioxygenases which act as 5mC oxidase, and replacement with unmodified cytosine (Zhao and Chen, 2014). In plants, this latter step of active DNA demethylation is primarily carried out by a small group of bifunctional DNA glycosylases that include ROS1, DME, DML2, and DML3 (Gong and Zhu, 2011). These remove the methylated cytosine base and create an abasic site, with the gap refilled by an unmethylated cytosine through a base-excision-repair pathway (Gong and Zhu, 2011).

### RNA Directed DNA Methylation

The RNA-directed DNA methylation (RdDM) epigenetic pathway is the primary mechanism by which plants mediate responses involving small RNAs, and is dependent upon the RNA polymerases Pol IV and Pol V, which are specific to plants, along with various accessory proteins currently being characterized (Matzke and Mosher, 2014). There is increasing evidence for involvement of RdDM in a wide range of developmental and physiological processes that include stress responses, pathogen defense as well as reproductive development (Boyko et al., 2010; Gutierrez-Marcos and Dickinson, 2012; Matzke and Mosher, 2014). This is in addition to the major role played in repression of subsets of transposons as well as protein coding genes, and the interplay between these in complex crop genomes has yet to be fully explored.

Within the nucleus snRNAs operate to repress epigenetic modifications such as 5mC and histone methylation directly at specific target sites, resulting in transcriptional gene silencing (TGS; Simon and Meyers, 2011; Matzke and Mosher, 2014). This involves processing of Pol IV transcripts within the nucleus and cytoplasm, and re-introduction into the nucleus, where siRNAs are able to facilitate targeting of Pol V nascent transcripts (Simon and Meyers, 2011). Recruitment of methyltransferase leads to *de novo* methylation of cytosines in each of the CG, CHG, CHH contexts, and although Pol V-mediated RdDM operates over many genomic regions, there appears to be a preference toward euchromatin, more recently acquired intergenic TEs, and genes containing TEs or other repeat sequences in their promoters and introns (Matzke and Mosher, 2014). A large proportion of RdDM targets are also modified by modified histone H3K9me, which can provide a feedback loop with DNA methylation to reinforce TGS.

### Biophysical Properties of 5mC

The methylation of cytosine (5mC) affects a wide range of DNA biophysical properties, with variation in the localized patterns of steric and conformational energy, as well as hydrophobic modifications to polarity (Hausheer et al., 1989; Wanunu et al., 2011). Together with electrostatic alterations that affect internal base pair dynamics, and variation in base stacking energy, these lead to variation in DNA flexibility (increased flexibility or bending propensity = decreased stiffness) and duplex stability. In particular, stacking energies between neighboring dinucleotides in DNA are represented by elastic force constants that contribute both to DNA flexibility and helical opening (Severin et al., 2011). In 5mC, these effects appear to be specifically associated with molecular polarizability of the pyrimidine, which increases the base stacking energy and reduced flexibility (Norberg and Vihinen, 2001; Acosta-Silva et al., 2010). This is due in part to the protrusion of the hydrophobic methyl group into the major groove, which alters the steric arrangement and local charge environment (Song et al., 2013). The base stacking interactions can generate local distortions in DNA (Acosta-Silva et al., 2010; Yusufaly et al., 2013) and inhibit CG:CG step overtwisting, which in turn decreases flexibility (Yusufaly et al., 2013).

In addition to DNA flexibility, stacking energies also contribute to the cooperative melting associated with the DNA helical-coil transition that is observed both in naked form (Anselmi et al., 2000) as well as within protein complexes (Perez et al., 2004), with helical stability also proportional to the local cation environment (Yakovchuk et al., 2006). Atomic force experiments and molecular dynamics simulations suggest that the contribution of 5mC to increased cooperative DNA helical stability may also depend on methylation level and sequence context, with perhaps more significant effects on mechanical stability and relative stiffness (Severin et al., 2011). Independent experimental evidence based on high resolution melting has also shown that 5mC confers increased helical stability compared with unmodified C (Rodriguez Lopez et al., 2010; Wanunu et al., 2011).

These contributions of 5mC to increased DNA duplex stability and reduced flexibility also appear to affect some aspects of nucleosome positioning, as well as the ability of nucleotide sequences to wrap around the histone complex (Dantas Machado et al., 2015). A picture that is emerging from recent plant whole genome methylome and nucleosome positioning studies (Pennings et al., 2005; Chodavarapu et al., 2010; Gelfman et al., 2013) suggests a discontinuous variation of 5mC in nucleosomal regions.

### H3.3 Distribution

Histone H3 is a substrate that provides considerable molecular complexity in terms of epigenetic marks for most eukaryotes, having two major variants, H3.1 and H3.3, as well as accommodating a range of post-translational modifications in the N-terminal amino acid residues. In *Arabidopsis*, H3.1 is enriched in silent areas of the genome, including those with the H3K27me3 and H3K9me3 modifications that contribute to transcriptional repression, as well as with DNA methylation (Stroud et al., 2012). In contrast, H3.3 has been shown to play a role in maintaining accessible chromatin (Jin and Felsenfeld, 2007), and is enriched in actively transcribed regions, particularly in the 3′ of *Arabidopsis* genes, where it is correlated with H3K4me3 and H3B ubiquitylation (Stroud et al., 2012).

### Histone H2A.Z Distribution

The histone variant H2A.Z is evolutionarily conserved, and often ∼60% identical to canonical H2A within a species, while being ∼80% conserved between species (Zlatanova and Thakar, 2008). The variant plays an important role in marking the epigenetic state of nucleosomes (To and Kim, 2013), and is preferentially localized toward the 5′ of genes in *Arabidopsis*, where it has been shown to be excluded from sites of heavily methylated DNA within actively transcribed genes (Zilberman et al., 2008). This inverse relationship between the H2A.Z and 5mC has been interpreted as providing a mechanism whereby H2A.Z protects DNA from cytosine methylation in euchromatic regions (Meneghini et al., 2003; Zilberman et al., 2008).

Increasing the wrapping of DNA around the core of H2A.Z containing nucleosomes can reduce the intrinsic fluctuations in DNA accessibility which facilitate transcription (Bowman and Poirier, 2015). Thus H2A.Z marked nucleosomes are often found in regions flanking TSS, and these provide a “molecular rheostat” for initiation of RNA Pol II transcription (Weber et al., 2014; Subramanian et al., 2015).

### H1 Linker

H1 histones interact with the linker DNA between adjacent nucleosomes, and cooperatively contribute to formation of the stable and compact 30 nm fiber (McBryant et al., 2010). Although the linker histones ensure compaction and stabilization of higher order chromatin, the variant forms also mediate variation in conformation and accessibility (Wong et al., 2007). It should be noted that linker H1 facilitates self-association of chromatin fibers at salt concentrations considerably lower than for nucleosomal arrays lacking H1 (McBryant et al., 2010). The stoichiometrical relationship between H1 and core nucleosomes has been shown to range from 0.5 to 1 in different tissues (Woodcock et al., 2006), with linker length conventionally described as a diagnostic feature of chromatin from different taxa and/or tissues (Woodcock et al., 2006).

Transient binding of H1 determines the trajectory of DNA entering and exiting the nucleosome (Bednar et al., 1998) by asymmetric binding of an entry or exit linker with the dyad axis (Brown et al., 2006), and constrains an additional 19–20 bp beyond the nucleosome core (Noll and Kornberg, 1977; Simpson, 1978). This is achieved primarily by neutralizing the negative charge of linker DNA, with the binding of the H1 C-terminal domain contributing to chromatin condensation (McBryant et al., 2010). More recently, additional roles for H1 histones have been uncovered, with the C terminal ends associated with molecular “hubs” that recruit proteins involved in accessing and modifying the chromatin fiber (McBryant et al., 2010).

Plants appear to have a wider range of H1 variants than animals (Over and Michaels, 2014), with many monocot and dicot species having at least one shorter variant that may be induced under drought stress (Jerzmanowski et al., 2000). For example, in *Arabidopsis*, H1.3 is drought inducible and has greater binding to chromatin (Ascenzi and Gantt, 1999). However, it should be noted that not all “drought inducible” H1 variants are associated with drought, and may contribute other functions during development (Over and Michaels, 2014). Depletion of the variants H1.1 and H1.2, along with removal of H2A.Z, is consistent with the global pattern of chromatin decondensation observed in *Arabidopsis* female megaspore mother cells (She et al., 2013).

Various lines of evidence have suggested a close interaction between linker H1 and ordered DNA methylation in plants (Wierzbicki and Jerzmanowski, 2005). For example, knockdown of H1 in *ddm1* mutants of *Arabidopsis* can lead to restoration of DNA methylation by RdDM (Zemach et al., 2013), suggesting that DDM1 is able to remove H1 to facilitate access to the DNA methylation machinery (Over and Michaels, 2014). Additional evidence has come from analysis of parent-of-origin imprinted loci in the MEDEA (MEA): DEMETER (DME) system, where DME acts as a 5mC demethylase and physically interacts with H1.2 (Rea et al., 2012). The same study has shown that *H1* mutants increase DNA methylation in maternal copies of *MEA* and *FWA* promoters in *Arabidopsis* endosperm. More recently, analysis of *h1.3* mutants has indicated that the absence of H1.3 can lead to significantly reduced stress-related DNA methylation, with this being most evident in the CHH context (Rutowicz et al., 2015). The requirement of H1.3 for a significant proportion of the DNA methylation associated with environmental stress suggests that this linker histone variation may facilitate chromatin accessibility in direct competition with the primary variants H1.1 and H1.2 (Rutowicz et al., 2015). More generally H1 depletion and DNA hypomethylation, along with H3K27me3 demethylation, appear to be key contributors to pluripotency that is facilitated by chromatin decondensation (Alatzas et al., 2008; He et al., 2012).

### Biophysical Properties of Histone Modifications

The complimentary roles of 5mC and H2A.Z associated with nucleosome stability may also be based on their respective biophysical and thermodynamic properties. H2A.Z has been shown to contribute to increased nucleosome stability compared with the canonical H2A, with structural and thermodynamic evidence for a more stable interface via the extended acidic path of the H2A.Z dimer and the charged tails of the (H3–H4)_2_ tetramer (Dechassa and Luger, 2012). These differences in electrostatic potential and size affect the interface with neighboring nucleosomes and other nuclear proteins (Chakravarthy et al., 2005), and can also contribute to compaction of the 30 nM fiber (Fan et al., 2002).

Sequence analysis of human H2A.Z and H2A-containing nucleosomes has also indicated a prominent association with DNA flexibility at nucleosome boundaries (Gervais and Gaudreau, 2009), with H2A.Z being slightly more rigid than corresponding H2A sequences. Moreover, a DNA flexibility model is able to predict the presence of H2A.Z bordering TSS (Gervais and Gaudreau, 2009). Biophysical studies have also indicated a decreased sensitivity of H2A.Z to ionic strength, with reduced organization of only ∼118 bp of core nucleosomal DNA compared with the canonical 147 bp (Doyen et al., 2006).

By adding a negative charge, phosphorylation of H1 generally has the effect of weakening the electrostatic interaction between H1 and DNA, thus increasing H1 mobility (Roque et al., 2008; Over and Michaels, 2014). Although the precise arrangement of H1 in relation to linker and nucleosome is still unclear (Woodcock et al., 2006), recent models suggest increased bending of DNA at the ends of the nucleosome core (Cui and Zhurkin, 2009).

## Nuclear Ionic Status and Chromatin Dynamics

### The Ionic Environment of the Nucleus

The nuclear envelope provides a boundary within which the genome resides and benefits from a distinct ionic environment buffered from the cytoplasm (Mekhail and Moazed, 2010; Van de Vosse et al., 2011). The two membranes of the nuclear envelope provide an interface, with discrete functions serving the nucleus and cytoplasm. In plants, the vacuole provides the primary store for inorganic ions (Seidel et al., 2013). However, it has become apparent that in all eukaryotes the perinuclear space between inner and outer nuclear envelope provides a store for calcium and other inorganic ions (Matzke et al., 2010), which may contribute to intracellular signaling (Draguhn et al., 1997), including rapid responses for maintaining selective homeostasis of ions such as K^+^ to sustain nuclear function (Wyn Jones and Lunt, 1967).

Although nuclear pores are not freely permeable to Na^+^ and K^+^ the outer membrane of the nuclear envelope contains distinct ion channel classes (Franco-Obregon et al., 2000), including K^+^ channels, which contribute to the Na^+^/K^+^ gradients between the perinuclear lumen, the nucleus and cytoplasm both in animals (Garner, 2002) and plants (Charpentier et al., 2008). Early X-ray microanalysis of oocytes demonstrated that only a portion of the K^+^ in interphase nuclei is in free ionic state, with the remainder being absorbed to the nuclear macromolecules, including DNA and histones (Cameron, 1985). As we shall see, the conformation of chromatin can be modulated by the electrostatic interaction mediated by ions such as K^+^. More generally, it is recognized that monovalent cations that are actively transported through nuclear channels are likely to play an important role in modulation of chromatin structure and gene expression (Garner, 2002).

### Chromatin Sensitivity to Cation Environment

The dynamic state of chromatin is subject to variations in the immediate thermal and ionic environment (Spadafora et al., 1979; Caño et al., 2006; Arya and Schlick, 2009). As we have seen, the genome exists in a crowded nuclear environment, embedded in chromatin and serviced by an array of RNA and protein molecules, with access to the read-only transcriptional capability being affected by thermodynamic and biophysical properties of the constituent macromolecules. The ionic environment of chromatin has significant effects on higher level chromatin conformation, with salt-dependent chain folding indicated by *in vitro* (Bertin et al., 2007) and electron microscope (Thoma et al., 1979) studies. As well as being guided by genomic sequence and distribution of epigenetic marks, global aspects of nucleosome assembly and disassembly appears to be dependent upon salt concentration, with the internal (H3–H4)_2_ tetramer of the nucleosome binding DNA more often at higher ionic strengths than the H2A–H2B dimer (Dechassa and Luger, 2012).

Evidence from molecular combing experiments, which generate a uniformly stretched array of DNA, has suggested that both Na^+^ and K^+^ inhibit binding of histone to DNA, whilst divalent cations significantly enhance binding, with Mn^++^ inducing condensation and aggregation of histone-DNA complexes *in vitro* (Liu et al., 2005). Thus increasing ionic strength is able to condense the 10 nm nucleosome fiber to form the 30 nm chromatin fiber as part of a reversible process arising from electrostatic repulsion overcoming nucleosome stacking interactions (Poirer et al., 2002). While an increase in monovalent cations above normal range may result in destabilization of interphase chromatin, low concentrations (10 mM) of divalent cations are able to condense chromatin (Visvanathan et al., 2013), possibly as a result of Mg^++^ mediating attraction between single negative charges along chromatin (Poirer et al., 2002).

Large scale sensitivity to the ionic environment is also apparent from the fact that attractive electrostatic interactions in chromatin can be screened by a high ion density with salt concentrations >100 mM (Poirer et al., 2002). In terms of visible phenotype, this has been found to lead to unfolding and expansion of chromosomes in *Notophthalmus viridescens* (eastern newt; Poirer et al., 2002). This may also account for the early observation that increasing the external supply of K^+^ up to 0.3 M in *Lolium temulentum* (ryegrass) had the effect of increasing meiotic chiasmata frequency at 30°C, although with little effect at 20°C (Law, 1963), and with little effect from Ca^+^.

Of more general significance, different lines of evidence from molecular modeling and *in vitro* studies now suggest that K^+^ and Na^+^ ions have distinct roles in condensation of DNA and chromatin, with recent *in vitro* evidence indicating that Na^+^ promotes the folding into 30 nm fibers in the presence of Mg^++^, whereas K^+^ limits this effect (Allahverdi et al., 2015). This appears to be due to the different binding behaviors of each ion to DNA, with K^+^ binding to the electronegative sites of DNA bases in the major and minor grooves, and Na^+^ interacting preferentially with the phosphate groups (Cheng et al., 2006). Moreover, there appears to be greater variation in the mobility of both water and ions in the K-DNA system than the Na-DNA system (Allahverdi et al., 2015). The consequences of this phenomenon for transcription, as well as the maintenance of Na:K ratios within the plant nucleus, have yet to be fully explored, but may have extensive ramifications for our understanding of how plant genomes harness and respond to the complex electrostatic environment within the nucleus.

### Ionic Variation in Plants

Cellular organisms expend a substantial proportion of their energy ensuring that the biochemical and other components within the cell are able to operate within boundaries of a relatively consistent ionic environment (Alekseeva et al., 2007). The internal concentration of specific mono- and divalent cations in plants appears to be under strong selection, with considerable variation observed across plant taxa based on data derived from tissue level assessment (Thompson et al., 1997; Broadley et al., 2004; Harada and Leigh, 2006). Whilst internal K, N, and P concentrations have been found to vary sixfold to ninefold between species of related angiosperm taxa (Thompson et al., 1997), concentrations of the divalent cations Mg^++^ and Ca^++^ appear much more variable, with a 49-fold variation in Ca^++^ and a 24-fold variation in Mg^++^. Most of this variance is allocated between monocot and dicot species (Thompson et al., 1997), and compares with P, where most of the variance is found at or below the species level.

Potassium (K^+^) is the most abundant inorganic cation in plants, representing up to 10% of dry weight (Watanabe et al., 2007), which is significantly more than required for optimal growth. Since the greater proportion is sequestered into the vacuole, most research attention has focused on its role as an osmoticant in the vacuole and cytosol, as well as an enzyme activator (Leigh and Wyn Jones, 1984), rather than its role in the nucleus. Much of the intra-specific variation appears to be under genetic control, with a greater than twofold variation in shoot K observed in *Brassica oleracea*, and quantitative trait loci (QTL) analysis suggesting a significant role for variation in K^+^ transporters (White et al., 2009). In the same species, levels of shoot Ca and Mg vary two and twofold to threefold respectively (Broadley et al., 2008), with a range of genes contributing to the uptake and homeostasis in leaf tissue (Hammond et al., 2011; Graham et al., 2014).

In general, plant species are able to exclude most of the salt (NaCl) present in soil solution, allowing about 2% to be transported in the xylem to shoots (Munns et al., 2006). Na^+^ severely inhibits most enzymes at levels >100 mM, and since more than 50 enzymes require K^+^ as a co-factor these are sensitive to Na^+^ and high Na^+^/K^+^ ratios (Tester and Davenport, 2003; Munns et al., 2006). Thus, while halophytic plants continue to grow at >250 mM, a number of crops, including rice, are compromised and die if soil salinity rises to 100 mM NaCl (Munns et al., 2006). Na^+^ toxicity is strongly associated with a plant’s ability to maintain uptake of K^+^, as well as the within plant distribution (Kader and Lindberg, 2005). Rice appears to have evolved in a low salt environment with plentiful supply of fresh water (Zong et al., 2007), and so yields start to decline at 30 mM Na^+^, compared with wheat at between 60 and 80 mM (Munns et al., 2006).

### External Ionic Conditioning and Epigenetic Variation

An early study using *Medicago* callus cultures indicated that while short term exposure to NaCl in had no effect on histone variant composition, it did lead to major increases in acetylation of H3.1, H3.3, and H4 (Waterborg et al., 1989). This was interpreted as an altered intra-nuclear ionic environment in the presence of salt, and possibly also representing an adaptive response in chromatin structure to permit chromatin function as Na^+^ increases (Waterborg et al., 1989). Since this study, a number of studies have detected an interaction between salt stress and histone modifications including both acetylation and methylation (Kim et al., 2015).

Although global DNA methylation is not significantly different between *Arabidopsis* shoots and roots, those regions of the genome that are differentially methylated tend to be preferentially (1.85×) hypermethylated in shoots (Widman et al., 2014), consistent with findings in rice (Karan et al., 2012). Within *Arabidopsis*, these sites in genes are primarily in the CG context, with a higher proportion at transcription initiation and termination boundaries. This also corresponds to a higher nucleosome density in these regions for the differentially transcribed genes, with the corresponding gene body being less nucleated. Overall it appears that roots tend to have a higher nucleosome density over genic regions and a more marked periodicity of DNA methylation (Widman et al., 2014). Moreover, genes with >10 × higher level of transcription in root tissue are more nucleosome-rich in the boundary regions compared with shoot tissue. The relationship between such observations and the prevailing intracellular thermal and ionic environments has yet to be explored. However, based on the findings in rice it has been suggested that the relative DNA hypomethylation observed in roots provides greater plasticity or preparedness for salt response genes (Karan et al., 2012).

DNA methylation has been shown to provide levels of environmental responsiveness in plant phenotypes, while providing some evolutionary flexibility in terms of heritability (Heard and Martienssen, 2014). Previous reviews have addressed a broad range of genetic and some epigenetic responses to extreme temperature or salt stress events (Madlung and Comai, 2004; Atkinson and Urwin, 2012; Mickelbart et al., 2015). Initial evidence that epigenetic mechanisms are more extensively involved in a range of plant responses to abiotic stress has come from reactivation of transgenes silenced by DNA methylation (Ito et al., 2011; Lang-Mladek et al., 2010). Thus, in tobacco the elevation of cold, salt, and metal ions all lead to hypomethylation of coding regions (Choi and Sano, 2007). The wider contribution of 5mC to management of stress responses has been revealed by the identification of large numbers of differentially methylated genomic regions, many with associated transcriptional changes, as a result of induction by stresses including bacteria and abiotic factors (Dowen et al., 2012). Although transposons may occur in these differentially methylated regions, such responses appear to be accompanied by up-regulation of 21-nt siRNAs, with many coupled to changes in transcription of the transposon itself and/or nearby genes (Dowen et al., 2012).

In soybean, salt stress has also been shown to induce hypomethylation, along with transcriptional activation of salt stress-induced TFs (Song et al., 2012). From evidence in rice, it appears that remodeling of DNA methylation may play a more general role in conditioning salt tolerance. For example, two salt tolerant genotypes have been found to have a significant level of hypermethylation compared with hypomethylation in two salt-sensitive genotypes (Feng et al., 2012). Although an independent study (Karan et al., 2012) did not find any specific methylation pattern associated with salt tolerant or susceptible genotypes under salt stress, there was a significant association with level of methylation and salt treatment in the shoot of four genotypes and in the root of two others. The authors concluded that many methylation changes associated with salinity were not directed (Karan et al., 2012), which may suggest a more generalized effect on the genome, particularly in the context of 5mCG gene-body methylation. This would be consistent with chromatin being in a more repressed (condensed) state, as well as the observation that stress leads to hypermethylation in satellite DNA at (non-genic) CHG sites within the halophyte *Mesembryanthemum crystallinum* (Dyachenko et al., 2006). It also appears consistent with the observation that mutations in components of the HDAC complexes reduce the ability of *Arabidopsis* to cope with salt and cold (Zhu et al., 2008; Chen et al., 2010), where more condensed chromatin may provide some protection against these stresses.

## Thermal Physiology and Contributions to Chromatin Conformation

### Thermal Physiology of Crop Plants

**FIGURE 2:**
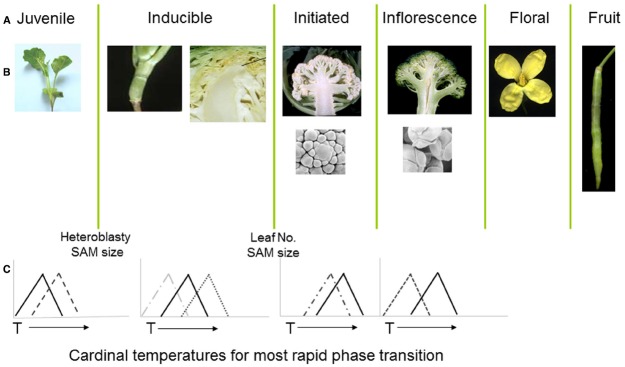
**(A,B)** Distinct developmental phases and phase transitions are clearly delineated in different genotypes of *Brassica oleracea*. Following floral initiation in cauliflower (var. *botrytis*) arrested development leads to proliferation of vegetative and inflorescence meristems, whereas in broccoli (var. *italica*) development is arrested at the later floral bud stage. **(C)** Each developmental phase may have a distinct cardinal temperature associated with an optimal rate of growth leading to the subsequent transition. X axes represent arbitrary temperature scale; Y axis relative growth rate.

A wide range of crop phenotypic traits are affected by thermal environment over multiplexed time-scales, from transitory responses through diurnal, circadian, and annual cycles (Bita and Gerats, 2013). Progress through the sequential developmental phase transitions associated with the detection, initiation, onset and progression of inflorescence development is co-ordinated by integration of environmental signals (Baurle and Dean, 2006; Huijser and Schmid, 2011; Figure 2). Detection of temperature is particularly important for the regulation and integration of signals contributing to onset of flowering, including the vernalization and photoperiod pathways (Huijser and Schmid, 2011). Many annual and perennial plants have evolved to fine-tune the sensing and integration of thermal signals (Patel and Franklin, 2009), with the ability to integrate periods of prolonged exposure below critical temperatures, together with thermal responsiveness, directly affecting crop performance (Luo, 2011; Robertson et al., 2013).

The cauliflower crop (*B. oleracea* var. *botrytis*) provides a useful demonstration of the consequences of temperature variation at different phases of development, with each phase transition typically sensitive to a genotype-specific optimal temperature range (Figure 2). Following seedling emergence, the plant remains in a vegetative juvenile phase during which it is unable to detect signals to initiate floral development (Wurr et al., 1994; Guo et al., 2004). The duration of this phase is sensitive to ambient temperatures (Wurr et al., 1995) with considerable variation dependent upon genotype (Fellows et al., 1999; Wurr et al., 2004). In most cultivars, including non-winter types, the plant undergoes a vernalization phase during which the vegetative apical meristem is responsive to accumulated thermal units in a range around a “cardinal” temperature optimum, with considerable genotypic variation also associated with this cardinal temperature (Wurr et al., 2004).

The developmental program in cauliflower leads to a proliferation of vegetative and inflorescence meristems to form the harvested curd (Smith and King, 2000), which is also sensitive to temperature variation (Wurr et al., 1990; Rahman et al., 2007). Radiation of the cauliflower and related broccoli crops from their centre of diversity in Italy (Massie et al., 1999) has resulted in cultivars containing alleles able to provide distinct cardinal temperature optima for length of juvenile period, vernalization, floral initiation, curd, and inflorescence initiation (Irwin et al., 2012). For some genotypes, high temperatures at the curd arrest stage may lead to the development of bracts, with a reduction in marketable quality (Kop et al., 2003), whilst others grown at temperatures below an optimal range may develop an undesirable “ricey” phenotype (Fujime and Okuda, 1996), where the arrested floral meristems progress to a later stage in development. This diversity of responses and phenotypic consequences are indicative of sophisticated gene regulatory mechanisms that are able to manage complex thermal signals in the context of distinct phases of development.

### Chromatin Responses to Thermal Variation

A range of properties associated with the chromatin macromolecular complex are affected by thermal variation. Thermal fluctuations are able to induce partial unwrapping of DNA from nucleosomes *in vitro*, and introduce twist or loop defects into the DNA wrapped round the core particle, resulting in repositioning in relation to the DNA (Blossey and Schiessel, 2011). Increased affinity (free energy, or enthalpy) due to association of histone proteins with DNA may also contribute to nucleosome translational or rotational positioning (Lowary and Widom, 1997), while relatively small quantities of free energy appear sufficient to precipitate association/disassociation with histone H1 (Rippe, 2012). In the absence of ATP-dependent DNA remodeling complexes, translational sliding of nucleosomes along DNA is temperature dependent, with repositioning taking place relatively slowly, at a rate of a few hours per 200 bp (Blossey and Schiessel, 2011). More generally, a range of histone modifications have been found to be associated with the heat stress response (Kim et al., 2015).

H2A.Z occupancy, especially at the TSS+1 nucleosomes of temperature-induced genes, has been shown in *Arabidopsis* to decrease with temperature, independent of transcription (Kumar and Wigge, 2010), so that when H2A.Z deposition is prevented, plants have a constitutive warm temperature response. Thus the canonical H2A nucleosomes do not contribute to unwrapping of DNA from the nucleosome in response to temperature, while H2A.Z nucleosomes become increasingly accessible to RNA Pol II as temperature rises. Where gene transcription decreases with temperature this may be due to H2A.Z providing greater access to binding of repressors at these loci, or by facilitating *de novo* DNA methylation (Kumar and Wigge, 2010).

### Involvement of H2A.Z in Thermosensory Flowering

The autonomous flowering pathway was conventionally regarded as being independent of environmental signals such as photoperiod. However, mutant analysis has demonstrated that genes of this pathway are also directly involved in mediating the effects of ambient temperature (Blázquez et al., 2003), with consequent effects on the expression of *FLOWERING LOCUS T* (*FT*), the mobile integrator gene of the floral pathway.

The mechanism by which the *FT* locus mediates the thermosensory flowering pathway (Halliday et al., 2003; Balasubramanian et al., 2006) is now being unraveled. It appears that while H2A.Z is enriched in the promoter region of *FT*, it is depleted at higher temperature, providing an explanation for acceleration of flowering in *Arabidopsis arp6* mutants deficient in H2A.Z (Kumar and Wigge, 2010). The chromatin modification that results from the heat-induced removal of H2A.Z from nucleosomes provides access to the *FT* promoter by the bHLH TF PIF4 (Kumar et al., 2012). In terms of control logic, H2A.Z is able to provide a genome-wide mechanism that is directly and rapidly coupled to temperature, and thus facilitate fine-tuning of phenotypic plasticity in response to environment.

This generic mechanism appears conserved in *Brachypodium*, a close relative of the major Pooideae grain crops, where H2A.Z nucleosomes appear responsible for the observed increase in thermal sensitivity of endosperm compared with vegetative tissue in the major monocot grain crops (Boden et al., 2013). Notably, H2A.Z nucleosome occupancy was more responsive to increases in ambient temperature in grain reproductive tissues, and correlated with the sensitivity to increased ambient temperature during early maturity. Thus the genomic organization of H2A.Z in *Brachypodium* results in limited impact of temperature on the phase transition from vegetative to reproductive stage, whilst retaining sensitivity at grain filling—a major contributor to yield in temperate grain crops. Perturbing the deposition of H2A.Z was found to be sufficient to mimic the effects of a warm temperature environment on grain development (Boden et al., 2013).

### Temperature Effects on RdDM

Temperature also appears to play a role in mediating RNA silencing (Szittya et al., 2003; Romon et al., 2013; Zhong et al., 2013; Liu et al., 2015). Post transcriptional gene silencing (PTGS) is characterized by accumulation of snRNAs, targeted degradation of mRNAs and DNA methylation of target genes. This can be inhibited in *Arabidopsis* by increasing growth temperature from 22 to 30°C (Zhong et al., 2013), and inherited through meiosis, affecting DNA methylation status as a result of exposure to higher temperatures in the previous generation. The release of PTGS appears to be due to a reduction in the formation of dsRNA required for production of siRNAs in the RNA silencing pathway, where the temperature increase reduces abundance of SUPPRESSOR OF GENE SILENCING 3 (SGS3). When over-expressed, SGS3 can reverse the warmth-induced inhibition of siRNA biogenesis and so reduce the transgenerational epigenetic memory (Zhong et al., 2013). Moreover, temperature induced release of sense transgene-mediated PTGS is dose dependent and stochastic between 24 and 28°C, but becomes deterministic at 30°C, with associated variation in warmth-induced DNA methylation within the target transgenes.

Basal heat tolerance in Arabidopsis also involves the RdDM pathway (Popova et al., 2013), with consequences for transcription and epigenetic regulation of transposons. *Arabidopsis* plants defective in either *NRPD2*, a subunit of RNA Pol IV and V, or in *HDA6*, an Rpd3-type histone acetylase, are hypersensitive to heat exposure, and these genes have independent roles in transcriptional reprogramming in response to temperature stress (Popova et al., 2013).

### Thermal Memory

Detection of accumulated thermal units or growing day degrees above or below a threshold has been resolved to major QTLs (Sadok et al., 2007; Bogard et al., 2014; Sánchez-Pérez et al., 2014), even in complex crop genomes such as *Brassica napus* (canola, rapeseed), where candidate genes at such loci appear to account for differences between over-wintering and summer crop types (Wang et al., 2012; Nelson et al., 2014). Natural flowering responses in *Arabidopsis* have also been localized to the *cis*-regulatory regions of the *FT* locus (Schwartz et al., 2009b). In *B. napus*, six copies of *FT* appear to have contributed to more complex mechanisms of floral regulation and niche adaptation compared to *Arabidopsis* (Wang et al., 2012) with promoter analysis (Wang et al., 2012) indicating that one copy (*FT*-C2) has been repressed by transposon insertion, with high levels of 5mC in both *B. napus* and the ancestral *B. oleracea*. Meanwhile the *FT*-A7/C6 homologs are specifically silenced in winter type *B. napus*, but abundantly expressed in spring type cultivars under vernalization-free conditions.

In *Arabidopsis*, dissection of the molecular mechanisms underlying vernalization has uncovered the role of epigenetic marks, particularly the polycomb-mediated additive effect of histone modifications, which under cold conditions regulate silencing of the flowering repressor *FLOWERING LOCUS C* (*FLC*, Sheldon et al., 2009; Romera-Branchat et al., 2014). Subsequently, it has been found that long non-coding RNAs (lncRNAs) COOLAIR (Swiezewski et al., 2009) and COLDAIR (Heo and Sung, 2011) are embedded within the *FLC* locus, and also induced during vernalization in *Arabidopsis* by periods of cold. COLDAIR has been proposed to recruit the H3K27me3 mark to the *FLC* gene, thus contributing to *FLC* repression (Heo and Sung, 2011), whereas some alternatively spliced isoforms of COOLAIR may contribute to activation of *FLC* (Csorba et al., 2014; Romera-Branchat et al., 2014).

### Temperature Conditioning and Epigenetic Variation

An early study by Burn et al. (1993) found that cold temperatures lead to hypomethylation in *Arabidopsis* and a *Nicotiana* cell line. Subsequently Finnegan et al. (1998) studied the effect of imbibing *Arabidopsis* C24 seed for 4 weeks at 8°C, and detected a large (86%) albeit transitory effect of hypomethylation in mature leaves compared with untreated controls at the same stage of development. These phenomena appeared to be reversible, as after 7 days growth at 22°C DNA methylation in seedlings developed from the vernalized controls was comparable to those of control seedlings. Cold stress has since been found to lead to genome-wide demethylation in maize seedlings (Steward et al., 2002), while growth of *Antirrhinum majus* in low-temperature conditions results in hypomethylation of the transposon *Tam3* (Hashida et al., 2006). A related phenomenon is also observed with heat-stress, where inducible alterations in endogenous loci generally lead to hypomethylation of retro-elements, with depression of transcription along with transient changes in nucleosome density (Lang-Mladek et al., 2010; Pecinka et al., 2010; Ito et al., 2011).

Histone methylation at H3K27me3 has also been shown to decrease gradually during cold exposure in two *Arabidopsis* cold-responsive genes *COR15A* and *ATGOLS3* (Kwon et al., 2009). It appears that in this case gene activation leads to removal of H3K27me3 and that this mark is able to be inherited quantitatively, providing a memory of recent transcriptional activity.

## A Speculative Framework: Electrostatic and Epigenetic Interactions within the Plant Nucleus

Our current understanding of the direct involvement of ionic and temperature variation on chromatin structure and transcription is fragmentary. However, it is clear that some key biophysical properties of chromatin components and epigenetic marks are affected by electrostatic and thermal interactions, and these more fundamental observations are starting to align with observations at cellular and whole organism level. In order to unravel these relationships, experimental approaches need to distinguish between direct effects and those mediated by signal transduction pathways that sense variation in external ionic or temperature environment and are then apparent at the level of epigenetic modifications.

Few studies have systematically evaluated the ensemble of interactions that place the ionic and thermal environment, the biophysical attributes of 5mC, H2A.Z and other epigenetic marks in the context of chromatin dynamics and genomic regulation (McClung and Davis, 2010). Thus, at present there is no cohesive model that takes into account the contribution made by each of the distinct epigenetic marks to chromatin conformation, and the ability of plants to maintain complex genomic regulation under fluctuating external environmental conditions. However, from the information presented in this review it is clear that temperature and ionic conditions both play an important role in determining the biophysical behavior of histone and DNA macromolecules, their interaction in forming nucleosomes, and in higher order chromatin conformation.

Taken together, the various lines of evidence outlined here appear to be internally consistent in describing contributions to accessible versus inaccessible chromatin. The framework that emerges is based on what appears to be a cohesive set of interactions at molecular, biophysical and electrostatic level between the various components that affect chromatin conformation and dynamics. This is represented in a simple schematic (Figure 1) that outlines the behavior of the key components of DNA, histones, and nucleosomes in the context of epigenetic marks and ionic environment within the plant nucleus. From this set of interactions, it is possible to speculate that within plant nuclei, general and localized ionic homeostasis plays a significant important role in maintaining chromatin conformation, whilst maintaining complex genomic regulation involving specific patterns of epigenetic marks.

The contributions of 5mC to local DNA stability and reduced flexibility appear to be consistent with the association of 5mC with stable, more ordered nucleosomes and localized transcription, with denser methylation leading to tighter chromatin and gene repression. The complementary contributions of 5mC and H2A.Z to less accessible chromatin is consistent with their observed relative mutual exclusivity in chromatin (Zilberman et al., 2008), and H2A.Z providing the transcriptionally responsive mark in response to external temperature (Kumar and Wigge, 2010).

Complex electrostatic interactions within the nucleus contribute to the condensation state of chromatin, with the localized net charge state of the interaction between DNA and histone affecting position and stability of nucleosomes. Post-translational histone modifications play a major role, although it is currently unclear how these may be affected by the proposed differential roles played by Na^+^ and K^+^ in chromatin condensation (Allahverdi et al., 2015). The latter findings have yet to be incorporated into our understanding of ionic variation in plants, and the dearth of knowledge about the ionic environment of plant nuclei, although Na^+^ and K^+^ gradients are observed within some eukaryote nuclei (Garner, 2002). Given the huge resources devoted to K fertilizer use of around 30 mt per annum (Timilsena et al., 2014) and concerns about salinization of cultivated land, it would seem timely to explore these phenomena in more detail at a molecular level to understand mineral ion availability in the nuclei of crop plants.

Whilst tentative, this framework provides scope to develop experimental approaches to understanding in greater detail the internal environment of plant nuclei. It is hoped that this will generate a deeper understanding of the molecular mechanisms underlying genotype × environment interactions that may be beneficial for long-term improvement of crop performance in less predictable climates.

## Concluding Remarks

A high proportion of crop traits exhibit quantitative inheritance, many with relatively low penetrance. For example, in rapeseed hundreds of significant environment-specific QTL have been identified for yield and other traits in a “fixed” segregating population of homozygous lines grown in 10 environments, with relatively few coinciding in multiple environments (Shi et al., 2009). In many cases the extensive genotype × environment interactions associated with crop yield traits has limited the ability to identify underlying genes. However, it is now apparent that QTL may also be accounted for by changes in DNA methylation status, with, e.g., 60–90% of heritability for the complex traits of flowering time and primary root length being detected in epiRILs of *Arabidopsis* (Cortijo et al., 2014). It is worth noting that in *Arabidopsis*, spontaneous transgenerational epiallelic variation can occur at a rate 10^3^ times higher than the genetic mutation rate (Becker et al., 2011), with hypermethylated alleles associated with siRNA production and TGS (Schmitz et al., 2011). This stochastic generation of epialleles has the potential to alter transcriptional behavior and generate novel phenotypic variation subject to selection. Thus formation of random epialleles mediated by RdDM may be of more significance than genetic variation (Matzke and Mosher, 2014). For crop breeding, there are clear indications that such variation needs to be under active selection, and attention given to maintenance of germplasm to ensure that epigenetic plasticity is hard-wired into new cultivars.

At present we have only a partial understanding of how the various epigenetic components confer dynamic functional information content in the context of the ionic and thermal environment of the nucleus. It is becoming clear that most regions of complex eukaryotic genomes play some role in gene regulation (Haudry et al., 2013). However, there is a need for systematic analysis of the relationships between plant genome complexity, the known taxonomic variation in ionic composition, the distribution of epigenetic marks and measures of genome ruggedness or plasticity. For example, does the relatively compact genome of rice, with less scope for redundancy in epigenetic regulation, also contribute to its inability to manage more extreme and complex abiotic stresses?

The ability of plants to accommodate fluctuations in thermal and ionic environment is an essential fitness attribute and a key determinant for crop performance, and requires a deeper understanding of the interactions at intra-cellular and intra-nuclear level, including those with epigenetic marks and processes. The availability of comprehensive tissue-specific epi-genome, nucleosome and snRNA datasets will contribute to more comprehensive models of interactions between genome organization, chromatin dynamics, and epigenetic signaling systems. These can help provide new tools and approaches for breeding selection and agronomic management of crops able to perform in changeable environments.

### Conflict of Interest Statement

The author declares that the research was conducted in the absence of any commercial or financial relationships that could be construed as a potential conflict of interest.
